# Design Considerations to Improve Charpy Instrumented Strikers

**DOI:** 10.6028/jres.125.010

**Published:** 2020-03-16

**Authors:** Nicholas Vlajic, Ako Chijioke, Enrico Lucon

**Affiliations:** 1 National Institute of Standards and Technology, Gaithersburg, MD 20899, USA; 2 National Institute of Standards and Technology, Boulder, CO 80305, USA

**Keywords:** Charpy, dynamic force, fracture mechanics, instrumented Charpy testing, instrumented striker

## Abstract

Instrumented impact testing allows the applicability of conventional Charpy tests to be extended toward assessing mechanical properties such as dynamic fracture toughness and dynamic tensile properties. In this work, we present design considerations for engineering instrumented strikers for Charpy V-notch impact testing. Specific attention is given to the mechanical and geometric features, as well as the placement of strain gauges and corresponding bridge circuits for instrumentation. These design considerations are intended to make the sensitivity invariant to the location and distribution of impact forces. The concepts presented in this work were applied to an actual instrumented striker, which was then statically calibrated. Data from this calibration indicate that the device has good repeatability, shows a linear response, and is relatively insensitive to impact location.

## 1 Introduction

Traditionally, the energy absorbed in fracturing a Charpy V-notch test specimen is calculated by measuring the difference between the initial height of the pendulum and its final height after breaking the sample, with corrections for windage and friction. Although this approach has proved to be reliable and repeatable, it can only provide a scalar quantity in units of energy. Additional information can be obtained with a force-time signal acquired during fracturing of the sample. The force-time signal can be obtained by installing a force measuring instrument near the striker or, more commonly, instrumenting the striker with one or more strain gauges onto a portion of the device. These strain gauges are typically part of a bridge circuit that produces a change in measured voltage for a given strain, which is proportional to an applied force. Impact tests performed using an instrumented striker are denominated instrumented impact tests.

Instrumented impact testing is often considered to be a relatively recent technical development of Charpy testing, even though the earliest known paper dealing with force measurements during an impact test [1] actually predates the first pendulum machine publication [2] by 1 year. The analysis of an instrumented Charpy test consists of the determination of characteristic time, force, displacement, and absorbed energy values corresponding to general yield, maximum force, initiation of unstable fracture, arrest of unstable fracture, and test termination. These characteristic values have been used for several analyses, such as:

• calculation of dynamic fracture toughness in the ductile-to-brittle transition region (see American Society for Testing and Materials [ASTM] E1921, Annex A1 [3]) or in the fully plastic regime (see ASTM E1820, Annex A17 [4]);
• investigation of the flow and fracture behavior of nuclear pressure vessel steels [5];
• estimation of dynamic tensile properties, such as yield strength [6] and tensile strength [7]; and
• assessment of the proportion of ductile fracture surface (shear fracture appearance [SFA]) in Charpy tests, as an alternative to direct optical measurements (see ASTM E2298 [8]).

The focus of this work takes the instrumented approach to measuring Charpy absorbed energy and emphasizes modification of the design and instrumentation of the striker to improve measurements.

After instrumentation with strain gauges, the striker must be calibrated in order to convert the bridge voltage to units of force; here, two key calibration challenges arise. First, the same distribution and location of applied force that occur during a test should be applied to the striker during calibration [8, 9]. The sensitivity dependence upon the distribution and location of forces on the impacting surface of the striker from commonly used striker designs is well known, and deviations of the sensitivity can be as high as 10% [10–13]. This attempted replication of force characteristics is sought by installing an untested Charpy specimen surrogate in the force-application instrument (most often a universal testing machine) [9]. This sensitivity dependence is not only a source of error in the calibration, but also during Charpy tests, as both the applied force distribution and location can vary for a number of reasons listed in Refs. [11, 12]. The second key challenge pertains to the rate at which the force is applied [14]. Standards today allow the striker to be calibrated using statically-applied forces [8, 9]; however, when fracturing samples, the force is dynamic with impact force durations between 0.1 ms and 10 ms. Researchers have acknowledged that discrepancies exist between static and dynamic sensitivities, and they have developed a number of ways to compensate for the difference in static and dynamic calibrations, such as the dynamic force adjustment [15] or the compliance approach [16, 17].

A few studies have strived to design an “optimal” instrumented striker by adjusting striker geometry or strain gauge placement, which is engineered to address these challenges [11, 18, 19]. This work is similar to those studies in that further design considerations to improve the striker performance are presented.

Manahan and Stonesifer [11] summarized the two critical design objectives, namely, that the striker response be invariant with respect to location and distribution of forces acting on the striker, and that inertial effects (or dynamic effects) originating from vibrations of the striker be minimized (which is meant to reduce the differences between static and dynamic responses). The former objective is primarily a static engineering problem, while the latter requires dynamic analysis. However, the two problems are linked, and a trade-off exists between each objective. Specifically, as the strain gauges are moved closer to the striking edge, inertial effects are minimized, but the sensitivity variation to the location and distribution of applied forces becomes large [11].

A striker that has a poor static performance (*i.e.*, a device with a sensitivity that is dependent upon the location and distribution of forces) will also have a poor dynamic performance for the same reasons. The design considerations presented here primarily address the static sensitivity challenges and only consider certain aspects of the dynamic response, namely, that the bridge circuit does not have a large sensitivity to certain vibrational modes. Other dynamic calibration challenges, such as minimizing inertia effects, are not addressed in this work, as the device presented here was dynamically calibrated using SI (International System of Units) dynamic forces. The dynamic calibration of this device is not reported here. Thus, the dynamic calibration will address the differences between the static and dynamic sensitivities, while the design considerations will primarily mitigate the static challenges.

The design concepts here utilized a “sensing element," specifically, a flexible portion of the striker that deflects when subjected to an axial force. The idea of a sensing element is not new and has been used in the design of force transducers and other types of sensors, including instrumented strikers. We instrumented the sensing element by securing strain gauges in a configuration that produces a voltage response to primarily axial forces and has a low response to bending moments and non-axial forces. Additionally, this configuration yields a sensitivity that is relatively invariant with respect to the location of applied force. The key design features of the device are discussed in detail throughout this document; however, a summary of these features are as follows:

1. Mechanical covers, used to protect the strain gauges, do not interfere with the deflection of the sensing element.
2. Features near the sensing element are smooth in order to prevent sharp or discontinuous gradients in the strain field when impact occurs.
3. Based on the geometry and deformation of the sensing element, the strain gauge configuration has been determined in order to measure the average transverse axial strain over the surface (rendering the sensitivity insensitive to the impact location) and to cancel out the response of certain vibration modes of the striker (*e.g.*, bending motions of the striker). This can be achieved using several small strain gauges (as will be described below), or using a single, wide strain gauge on each face of the sensing element.

Lastly, we note that the design of this striker was aided by the use of finite element analysis. Finite elements were used to test the proposed configuration of strain gauges for performance before construction, but they were not used as part of an optimization routine. Rather, these results gave insights into the deformations of the striker, and they allowed us to test the proposed configuration of strain gauges before constructing a device.

## 2 Mechanical Design Considerations

**Fig. 1:**
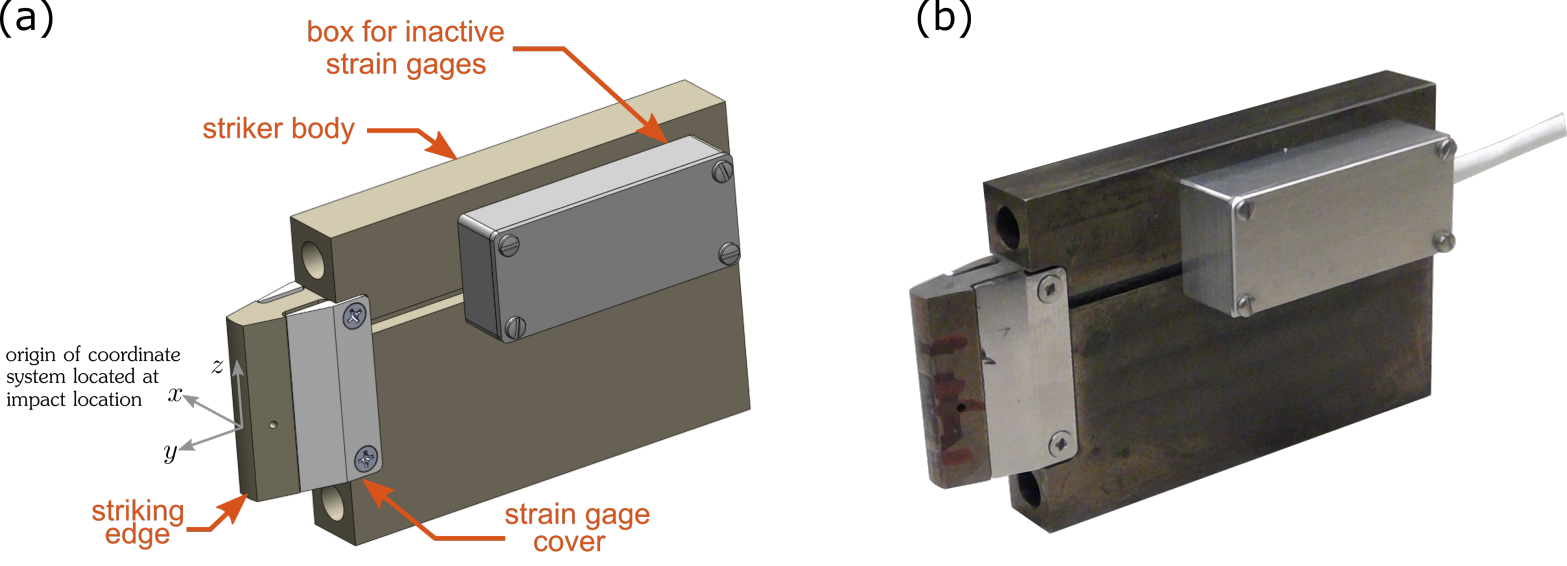
(a) Rendering of the instrumented striker used within this work. (b) Actual photograph of the device.

**Fig. 2:**
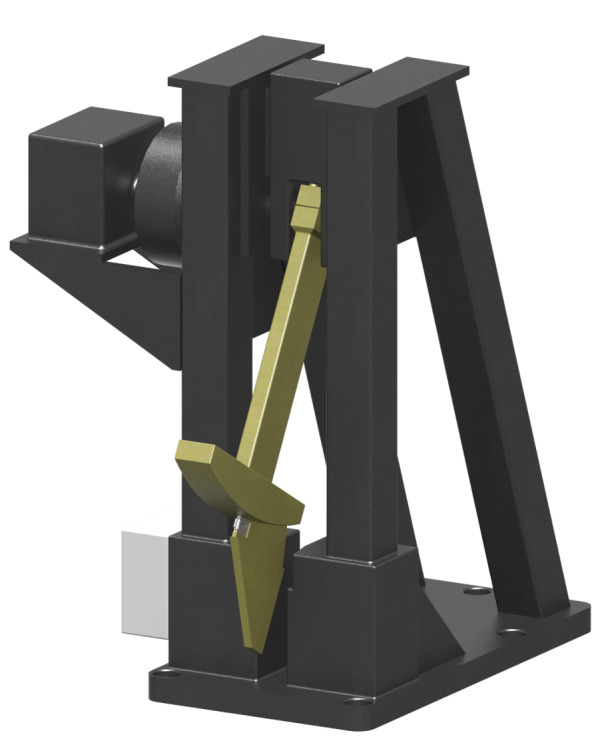
Rendering of the Charpy machine in which the striker presented in this study is embedded.

A rendering of the instrumented striker is shown in Fig. 1(a), while a photograph of the device is provided in Fig. 1 (b). Detailed dimensions of the device and specific placement of the strain gauges are provided in Section 7, Appendix A. This style of striker, namely, one designed for a machine equipped with a “Z-style" hammer, was selected because it is smaller in size and more symmetrical in shape than the strikers used in machines equipped with “U-style" hammers. A rendering of the Charpy machine used in this study is given in Fig. 2.^1^1 Certain commercial equipment, instruments, or materials are identified in this paper in order to specify the experimental procedure adequately. Such identification does not imply recommendation or endorsement by the National Institute of Standards and Technology (NIST), nor does it imply that the materials or equipment identified are necessarily the best available for the purpose. The strain gauges are mounted near the striking edge and are protected by a mechanical cover. The strain gauge bridge circuit (discussed in Sec. 3) requires inactive strain gauges that are necessary to complete the bridge circuit. These inactive gauges are secured to a piece of metal made from the same material as the striker body and covered in a box on the side of the striker. Finally, the output of the bridge circuit is connected to an amplifier/signal conditioner through a cable.

### 2.1 Material Selection

Presently, ASTM [20] and International Standards Organization (ISO) [9, 21] standards do not specify a striker material or minimum hardness; however, ISO 14556:2015 [9] does recommend a minimum hardness of 56 HRC (where HRC indicates Rockwell hardness scale C) for the support block in statically testing the striker. Common commercially available strikers have similar hardness values near 56 HRC. The one presented here was made from 350 maraging steel (Aerospace Materials Systems [AMS] 6515), which was heated to 900 *^◦^*C for 12 h and then air cooled. Based on this aging schedule, a hardness near 57 HRC is to be expected. The strain gauge covers were made from 316 stainless steel, while the box that covers the inactive strain gauges was made from 6061 aluminum. This material selection should not be necessarily considered optimal. The stainless steel covers and aluminum box are not subjected to large forces during a normal impact and do not necessarily need to be made from a hardened material; however, the cover and junction box could have been made from maraging steel for durability and robustness.

### 2.2 Geometry of the Striker

Current ASTM standards only specify dimensions related to the geometry of the striking edge of the striker [20], while ISO standards specify dimensions of the geometry of the striking edge back to the body of the striker [9, 21]. The current documented standards allow for flexibility in designing the remaining portions of the device. The striker presented here was designed to have a sensing element–an area that is intentionally flexible and serves as the force transducer. During calibration and use of the device, the sensing element must be able to deflect without interference. Moreover, the sensing element should be free of features that cause large stress concentrations (*e.g.*, sharp corners and holes), although it is not required that the strain field be uniform or constant within the sensing element. The striking edge of the striker was made in accordance with ASTM E23 [20], which specifies an 8 mm radius on the striking edge.

**Fig. 3:**
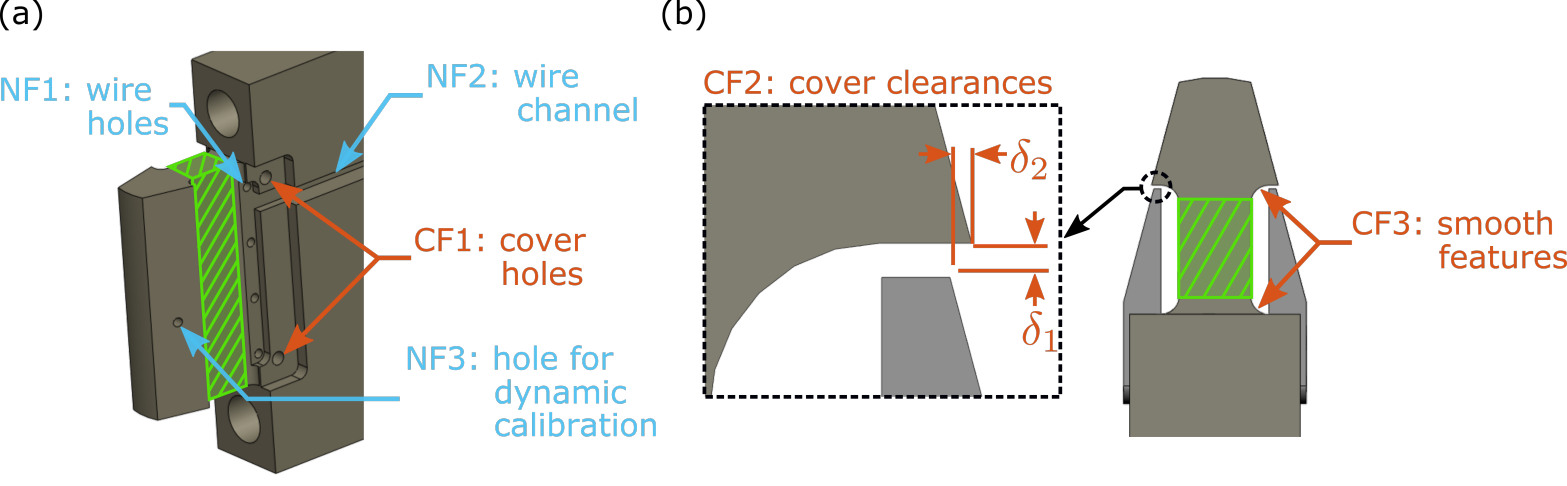
(a) Isometric view of the instrumented striker shown in Fig. 1(a) without the cover plates, and (b) top view of the instrumented striker with cover plates. The sensing element of the striker is highlighted in green cross-hatch (///). Features labeled CF are critical features (denoted in orange *•*), which enhance the performance of the device, while features labeled NF (denoted in light blue *•*) are considered features necessary for practical implementation. Clearances *δ*_1_ *≈* 0.2 mm and *δ*_2_ *≈* 0.13 mm are introduced to prevent the covers from interfering with the deflecting element and specimen, respectively.

Renderings of the striker with and without the covers are shown in Fig. 3, where the sensing element is highlighted in the cross-hatch area (also denoted by the color green ///). Features labeled CF1, CF2, and CF3 (also denoted by the color orange •) are considered critical features (CF) to enhance the performance of the device, while annotations labeled NF1, NF2, and NF3 (also denoted by the color blue •) are necessary features (NF) for the construction, but these are not considered critical with respect to performance.

The holes used to mount the cover were placed away from the sensing element as shown in CF1, so as to not create strain concentrations near the locations of the strain gauges. The covers then protrude and extend over the strain gauges, but they do not touch the sensing element or the striking edge as pointed out in CF2.

Although the covers can vibrate, their first natural frequency is higher than the frequencies generally of interest (up to 30 kHz). The clearance $\delta_{1}\approx 0.2$ mm ensures that the cover does not contact the striking edge during impact, while clearance $\delta_{2}\approx 0.13$ mm is an inset, so that the cover does not interfere with the specimen during fracture, especially in the case of very high-energy specimens, which can wrap around the device.

The four holes labeled NF1 serve to run wires for the strain gauges to the opposite side of the striker, while the channel (NF2) is an avenue by which the strain gauges can be connected to the remaining portions of the bridge in the box located near the end of the striker. The single hole on the impactor (labeled NF3) is used in one method of dynamically calibrating the striker, but the dynamic calibration itself is not discussed within this work. The holes labeled NF1 and NF3 are more than three diameters away from the sensing portion of the strain gauge and will have little effect on the strain field at the location of the strain gauges.

## 3 Instrumentation Considerations

When the striker impacts a specimen, a strain field is generated in the device. Typically, the strain is measured using a strain gauge bridge circuit. A single strain gauge or a small number of strain gauges on a sensing element will only produce changes in the bridge voltage that are proportional to strains in a localized area of the field. Measuring the strain of a single point (or small area) of a strain field will yield a sensitivity that is dependent upon impact location, as the strain field will vary depending upon the location where the specimen makes contact with the striker. For the same reason, the static calibration can have errors.

Furthermore, the strain gauge(s) may respond to certain vibrational modes of the device, which are not indicative of the force applied to the end of the striker. There were four objectives when selecting the placement of strain gauges on the sensing element. The objectives were to orient the gauges and construct a corresponding bridge circuit such that: (1) each strain gauge has an equal weight in the response of the circuit, (2) the strain gauges and corresponding bridge circuit give rise to a voltage proportional to the average strain over most of the area of the sensing element, (3) the response of the combination of strain gauges is most sensitive to axial forces (*i.e.*, forces in the y-direction), and (4) the response is relatively insensitive to the location of the applied force and to (nonaxial) vibrational modes of the striker itself.

**Fig. 4:**
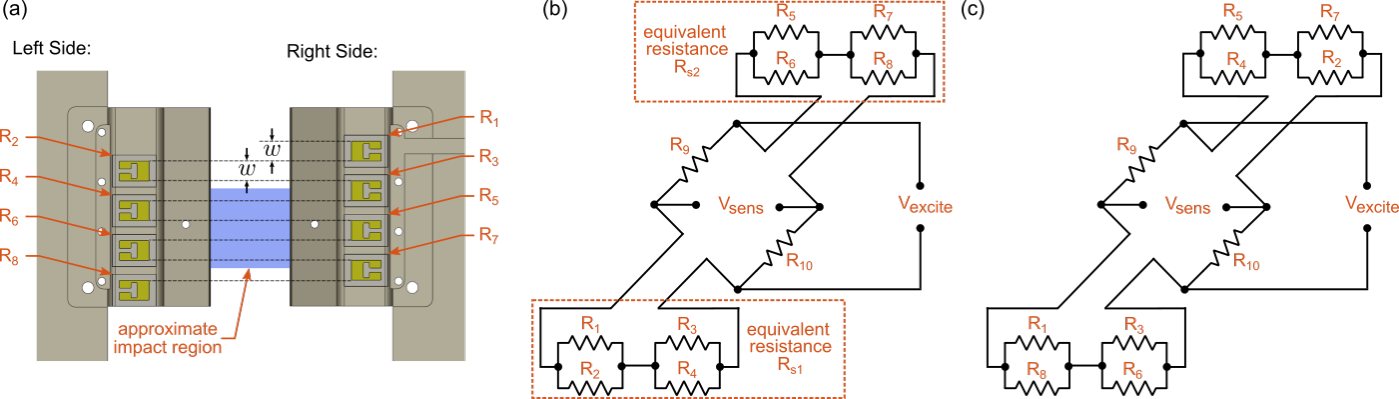
(a) Mechanical layout of the strain gauges, where the spacing between the strain gauge matrix edges is ideally equal to the width of the strain gauge matrix *w* to ensure complete, uninterrupted coverage in the vertical direction. (b) Bridge circuit corresponding to the strain gauge configuration shown in part (a). (c) Bridge circuit that is equivalent to the circuit shown in (b).

The configuration of the strain gauges on the sensing element is shown in Fig. 4 (a), while the strain gauge bridge circuit is shown in Fig. 4 (b). A second bridge circuit, shown in Fig. 4 (c), is mathematically equivalent to the one shown in Fig. 4 (b). Strain gauges R_1_ through R_8_ primarily measure the axial strain in the sensing element, while gauges R_9_ and R_10_ are inactive gauges that serve to complete the bridge. These inactive strain gauges are adhered to a piece of metal made from the same material as the striker, which is secured using room-temperature-vulcanizing (RTV) silicone, and housed in a box located near the rear of the striker; (see Fig. 1(a) and (b)). The same striker body material was selected as the host of inactive strain gauges for temperature compensation reasons, while the RTV silicone isolates the inactive gauges and material from mechanical vibrations.

The active strain gauges are staggered on the left and right sides to align the edges of each of the sensing elements to its corresponding neighbor on the opposite side. This configuration ensures continuity in the vertical direction of the sensing element. Effectively, these strain gauges average the strain in the axial direction over the vertical region of the sensing element.

The measured voltage ratio of the bridge circuit $V_{sens}/V_{excite}$ is given to be


$$\frac{V_{sens}}{V_{excite}}=\frac{R_{s1}R_{s2}-R_{9}R_{10}}{\left(R_{s2}+R_{10}\right)\left(R_{s1}+R_{9}\right)},\;$$

where


$$R_{s1}=\frac{R_{1}R_{2}\left(R_{3}+R_{4}\right)+R_{3}R_{4}\left(R_{1}+R_{2}\right)}{\left(R_{1}+R_{2}\right)\left(R_{3}+R_{4}\right)},\;$$

$$R_{s2}=\frac{R_{5}R_{6}\left(R_{7}+R_{8}\right)+R_{7}R_{8}\left(R_{5}+R_{6}\right)}{\left(R_{5}+R_{6}\right)\left(R_{7}+R_{8}\right)}.\;$$

The resistance of the $i^{th}$ resistor is given to be $R_{i}=R+\delta R_{i}$ (for i=1...8), where R is the nominal resistance, and $\delta R_{i}$ is the change in the resistance due to deflection. The bridge output variation $\delta V_{sens}$ is determined by substituting the expression for $R_{i}$ into Eq. (1), Eq. (2), and Eq. (3); and linearizing the $\delta R_{i}$ around zero. This results in the following variation of the bridge output:


$$\frac{\delta V_{sens}}{V_{excite}}=\left(\frac{1}{2R}\right)\left(\frac{1}{8}\sum_{i=1}^{8}\delta R_{i}\right)=\frac{1}{16R}\sum_{i=1}^{8}\delta R_{i}.$$

**Fig. 5:**
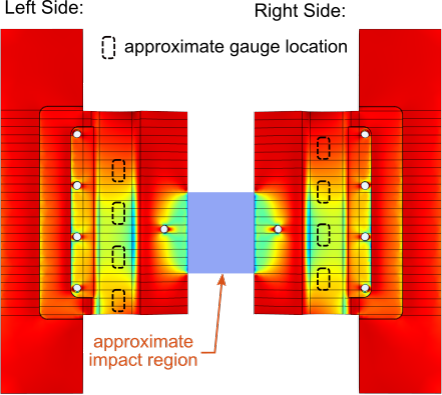
Static finite element analysis results, where dark blue represents areas of large compressive axial strain while red areas indicate strains close to zero. Approximate locations of the strain gauges are indicated in dashed, black lines.

From Eq. (4), it is clear that this configuration of strain gauges averages the strain over the sensing element. The effectiveness of averaging the axial strain over the sensing element is demonstrated in Fig. 5, wherein static finite element analysis results are shown for the instrumented striker. A 10 kN force was applied over the region shown in blue. The surface colors correspond to the normalized axial strain throughout the device. Blue and green areas indicate areas of maximum compressive axial strain, while red areas indicate little to no axial strain. Figure 5 illustrates the localized strain fields that are generated when a sample is in contact with the front of the striker. The response from a single strain gauge will vary depending on the impact location. Although the axial strain field will vary with respect to the applied force location, the summation of all the strain gauges should be largely invariant to location and only respond to the magnitude of the applied force. The proposed configuration attempts to average the strain over the web.

Additionally, the configuration was constructed in such a way that the bridge will theoretically not produce a voltage for pure bending motions about the z-axis (corresponding to deflections in and out of the page). For example, if the striker bends in the positive z direction symmetrically about the centerline, the odd-numbered strain gauges will see a compressive bending strain of


$$-\epsilon_{b}=\epsilon_{1}=\epsilon_{3}=\epsilon_{5}=\epsilon_{7},$$

while the even number gauges will see a tensile strain of equal magnitude as


$$\epsilon_{b}=\epsilon_{2}=\epsilon_{4}=\epsilon_{6}=\epsilon_{8}.$$

Given that all eight strain gauges have a similar gauge factor $G_{F}$, and noting that $\delta R_{i}/R=G_{F}\epsilon_{i}$, substitution of these resistance values into Eq. (4) shows that $\delta V_{sens}$ will be zero for these bending motions. Although these arguments have been demonstrated using a static bending strain $\epsilon_{b}$, they also hold for vibrational motions wherein the strain is periodic.

**Fig. 6:**
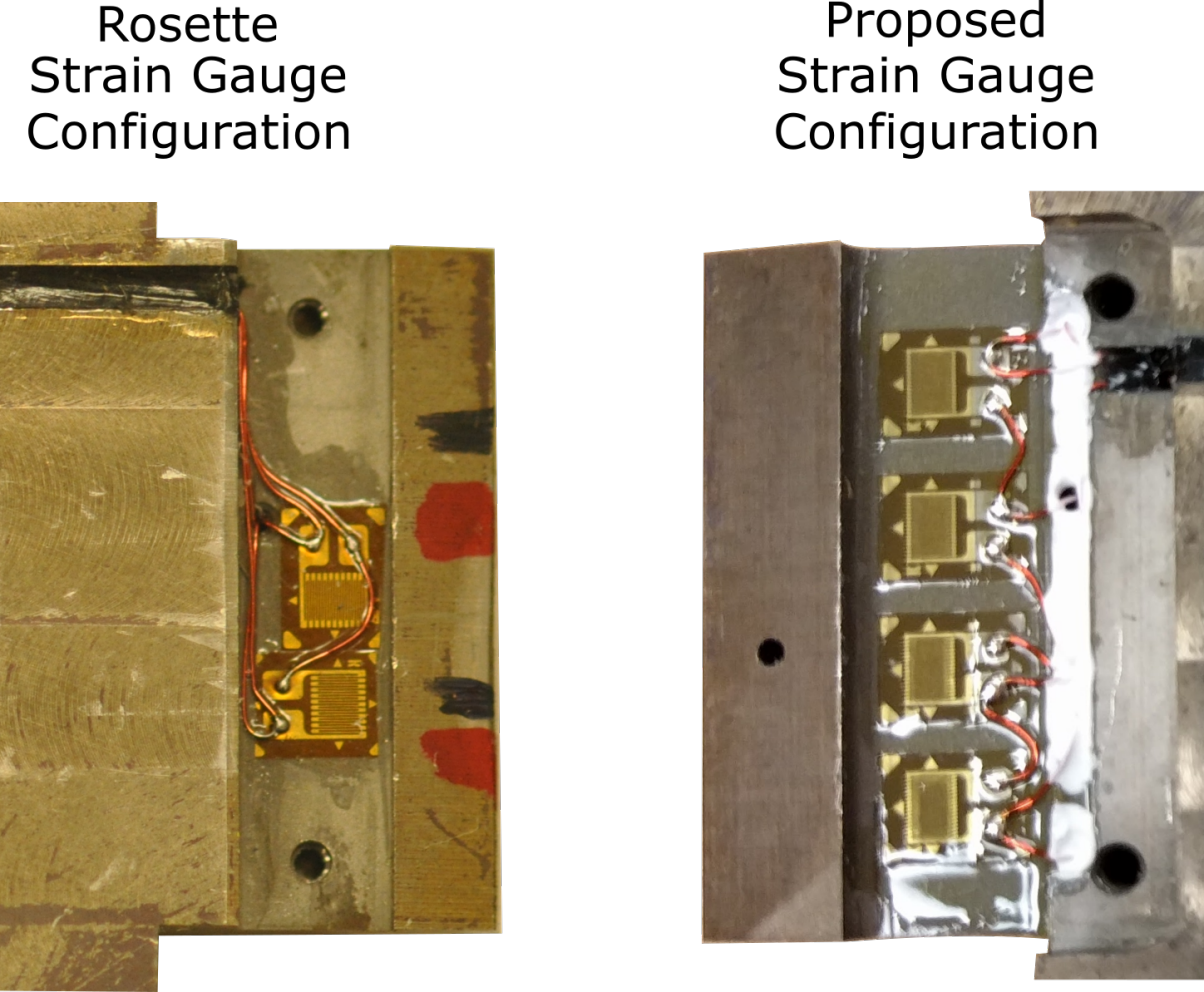
Two photographs of strikers designed at National Institute of Standards and Technology that compare a rosette strain gauge configuration to the proposed configuration. The red and black markings on the striker with the rosette configuration were used for statically calibrating the device.

Moreover, because the gauges are staggered between the left and right sides, the bridge circuit will have a small response if bending is not uniform or for torsional motions about the z-axis (*i.e.*, torsional motions wherein the top of the sensing element goes into the page and bottom comes out of the page, or vice versa). If the gauges were not staggered (*i.e.*, they were directly across from one another), the non-uniform and twisting deflections would then be nulled; however, the circuit would then have a sensitivity dependence on impact location. Alternatively, an odd-number of strain gauges can be used to null out twisting motions, but to the best of our knowledge, the bridge cannot be constructed in such a way that all gauges have an equal weight in the response of the bridge circuit. We also note that this circuit was designed so that each leg of the bridge has a nominal resistance of R=350   Ω. Resistors R_1_ to R_4_ and resistors R_5_ to R_8_ can be put into series, respectively. This would also require changing the value of resistors R_9_ and R_10_ to balance the bridge. Ideally, each side of the sensing element would have one wide strain gauge designed for sensing primarily in the y-direction (see Fig. 6). A strain gauge such as this could be lithographically deposited onto the striker.

## 4 Experimental Evaluation of Static Performance

### 4.1 Static Calibration Data

**Fig. 7:**
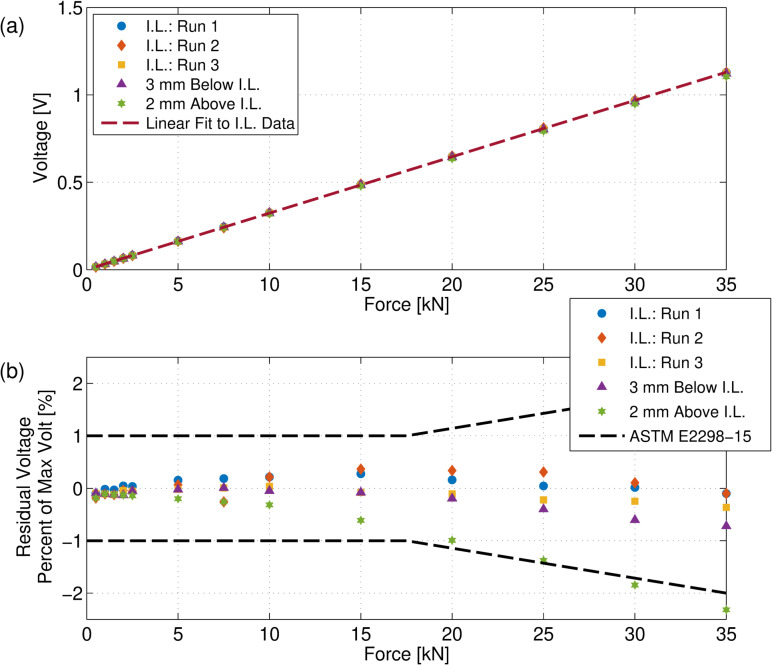
(a) Calibration data at the nominal impact location (I.L.) and data where the force was applied 3 mm below the impact location and 2 mm above the impact location. (b) Residual plot from a linear fit to the impact-location data, along with the allowable error specified by ASTM E2298-15 [8].

The device was statically calibrated using 13 force steps, with the force applied at the impact location and an amplifier gain of 100. The forces were applied using a universal testing machine that was equipped with an adapter that applied the force through an untested or broken Charpy specimen, in order to replicate the configuration that the striker would encounter during a test. A summary of the static calibration data is provided in Table 1. The force was applied to the impact location in three separate runs at the 13 different force values, after an initial loading up to 25 kN to “seat" the striker and produce an indentation in the support. Standard deviations were less than 1% for most of the force values tested, which indicates good repeatability for such a device. A plot of the data listed in Table 1 is given in Fig. 7(a), and a plot of the residuals from a linear fit is shown in Fig. 7(b). The linear fit was determined by fitting the mean data from three runs. The data indicate that the response of the device is quite linear and has a slope (*i.e.*, a sensitivity) of C=32.26 mV/kN, with a regression coefficient R=0.99999. The deviations of the data from the line were all less than 0.5%.

**Table 1:** Static calibration results of the instrumented striker with the proposed design considerations using a universal testing machine when applying force at the impact location, 3 mm below the impact location, and 2 mm above the impact location. The percent difference is the percent difference between the reading at the off-impact location and the mean of the readings when loaded at the impact location.

Force (kN)	Applying Force at Impact Location (I.L.)					I.L. - 3 mm Run 1 Percent (V) Diff. (%)		I.L. + 2 mm Run 1 Percent (V) Diff. (%)	
	Run 1 Run 2 (V) (V)		Run 3 (V)	Mean Std. Dev. (V) (V)					
0.5	0.016	0.015	0.016	0.016	0.0006	0.016	2.13	0.015	-4.26
1.0	0.033	0.032	0.032	0.032	0.0006	0.032	-1.03	0.032	1.03
1.5	0.049	0.048	0.048	0.048	0.0006	0.048	-0.69	0.048	0.69
2.0	0.066	0.065	0.065	0.065	0.0006	0.064	-2.04	0.064	-2.04
2.5	0.082	0.081	0.081	0.081	0.0006	0.081	-0.41	0.080	-1.64
5.0	0.164	0.163	0.162	0.163	0.0010	0.162	-0.61	0.160	-1.84
7.5	0.245	0.240	0.243	0.243	0.0025	0.243	0.14	0.240	-1.10
10.0	0.326	0.326	0.324	0.325	0.0012	0.323	-0.72	0.320	-1.64
15.0	0.488	0.489	0.484	0.487	0.0026	0.484	-0.62	0.478	-1.85
20.0	0.648	0.650	0.645	0.648	0.0025	0.644	-0.57	0.635	-1.96
25.0	0.808	0.811	0.805	0.808	0.0030	0.803	-0.62	0.792	-1.98
30.0	0.969	0.970	0.966	0.968	0.0021	0.962	-0.65	0.948	-2.10
35.0	1.129	1.129	1.126	1.128	0.0017	1.122	-0.53	1.104	-2.13

### 4.2 Sensitivity to Location of Applied Force

As noted above, the sensitivity to transverse (x and z-directions) impact location affects both the static and dynamic responses during calibration and use of the device. The device sensitivity dependence on the location of the applied force was determined by applying forces off of the nominal impact location, denoted by z=0 mm. The force was applied at z=-3  mm±0.5  mm, corresponding to 3 mm below the nominal impact location, and z=2  mm±0.5  mm, corresponding to 2 mm above the nominal impact location (see Fig. 1 for a coordinate system). The output voltage results from these off-impact-location forces are shown in Table 1 and in Fig. 7. Most of the absolute values of the differences between the response to the off-impact-location force application and impact-location force application were less than 2%. We note that these off-impact-location values are extreme in comparison to the repeatability of the impact location in a Charpy machine with regard to the nominal location. However, effects of off-impact-location sensitivity can be aggravated in actual impacts if the striker and sample first engage at a point. In other words, the striking edge of the striker and the specimen are not parallel when they first engage, resulting in a point-contact of the striking edge with the top or bottom portion of the sample. The slope of the fit line at the location 3 mm below the nominal impact-location was C=32.07 mV/kN, and for the location 2.0 mm above, it was C=31.58 mV/kN, which are comparable to the sensitivity at the impact location. We note that the deviation of the sensitivity from the nominal value is greatest for the location 2 mm above the nominal impact-location. This deviation could originate from small dimensional differences from nominal dimensions in the placement of the strain gauges or from non-uniformity in the fabrication of the device.

The same test of sensitivity dependence to impact location was also performed on an instrumented striker with a flexible element and a strain gauge rosette configuration, which is shown in Fig. 6. The rosette configuration on the side shown is a mirror image of the opposite side and was wired in a full-bridge configuration. This striker was fabricated with the previously mentioned design considerations (listed in Sec. 1). The comparison with this configuration was selected since strain gauge rosette configurations are commonly used in instrumenting engineering structures [22], as well as Charpy instrumented strikers. Two other strain gauge configuration recommendations on instrumented strikers that do not make use of a flexible element are given in ISO 14556:2015. The sensitivity of the design presented here changes by -0.59% and -2.12% of the nominal value for the -3 mm and 2 mm locations, respectively, while the sensitivity of the rosette device changes by -34.8% and 1.39% for the same locations. The rosette device has a large sensitivity to impact location, because the gauges are measuring a local strain, rather than an averaged strain.

### 4.3 Sensitivity to Distribution of Applied Forces

In order to test for changes in sensitivity due to changes in the distribution of forces, both strikers were also statically calibrated by applying forces to the striker through a 10 mm × 10 mm stainless steel unnotched specimen, a 8 mm × 8 mm stainless steel unnotched specimen, and 10 mm × 10 mm aluminum 6061 unnotched specimen. The different sizes and materials will create different stress and strain distributions on the striking edge, which could affect the sensitivity of the device. The static sensitivity of the two strikers was determined by averaging three data points at 14 different force values between 0.5 kN and 35 kN. The aluminum samples started to plastically deform at much lower force values than the steel specimens, so the striker’s sensitivity was determined by averaging three points at 18 force values between 0.5 kN and 10 kN. A summary of the sensitivities is shown in Table 2, where the sensitivity dependence on force distribution is shown for two ranges of applied forces, namely, the 0.5 kN to 10 kN range and the 0.5 kN to 35 kN range. The proposed configuration shows smaller changes in sensitivity for the 8 mm and 10 mm stainless steel samples for both ranges of data, and it has a comparable sensitivity change to the rosette configuration for the 10 mm aluminum sample. Other commonly used striker designs have shown sensitivity changes as high as 10% while varying the distribution and location of forces on the striking edge of the striker [10-13].

**Table 2:** Summary of the device sensitivity when subjected to off-center loading and different force distributions, generated by using stainless steel (SS) and aluminum (Al) unnotched specimens. For the force distribution measurements, the sensitivities were determined using a force amplitude range of 0.5 kN to 10 kN and a larger force amplitude range of 0.5 kN to 35 kN. All sensitivities listed have units of mV/kN.

	Nominal	LOCATION		DISTRIBUTION (10 kN)			DISTRIBUTION (35 kN)	
	Sensitivity	I.L. - 3 mm	I.L. + 2 mm	SS	SS	Al	SS	SS
	(mV/kN)			10 mm	8 mm	10 mm	10 mm	8 mm
Rosette	66.56	43.39	67.49	67.85	71.64	65.41	65.07	68.56
Configuration	% change	-34.8	1.39	1.93	7.62	-1.73	-2.25	3.00
Proposed	32.27	32.07	31.58	32.61	33.17	32.89	32.32	32.73
Configuration	% change	-0.593	-2.12	1.07	2.81	1.96	0.175	1.43

## 5 Experimental Evaluation of Dynamic Performance

### 5.1 Comparisons of Energy

**Fig. 8:**
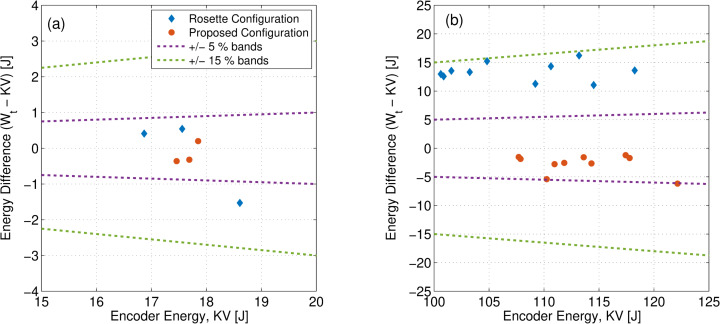
Comparison of the difference in encoder energy (*KV*) from the instrumented energy (*W_t_*) for (a) low-energy (LL 137) and (b) high-energy (HH 107) specimens. Bands represent 5% and 15% differences per ASTM E2298-15 [8].

Both strikers, namely, the proposed configuration and the rosette configuration, were used to determine the energies required to break Charpy specimens. Three low energy specimens (lot LL 137) and 10 high-energy specimens (lot HH 107) were tested for each striker. Results from these tests are shown in Fig. 8.

Standard ASTM E2298-15 [8] specifies the difference in encoder and striker energies to be less than ±15% of the encoder energy or 1 J, whichever is larger. For reference, the ±15% and ±5% difference bands are also shown in Fig. 8. Both striker configurations conform to ASTM E2298-15 for low- and high-energy specimens. However, the agreement between encoder and striker energies is better for the proposed configuration than the rosette configuration, as seen in Fig. 8. The data presented in Fig. 8 suggest that the proposed configuration has better dynamic performance than the traditional rosette configuration.

For the lower energy samples, the average absorbed energy was 17.7 J *±* 0.40 J (where the uncertainty represents twice the standard deviation) for the proposed configuration; and 17.7 J *±* 1.75 J for the rosette configuration. Large numbers of samples were characterized in National Institute of Standards and Technology Interagency/Internal Report (NISTIR) 8145 [23], wherein characterization data were gathered with five different NIST Charpy machines. NISTIR 8145 determined the energy to be 17.78 J *±* 1.60 J (see Table 14 in Ref. [23]), which compares well with values obtained by both strikers here, although the proposed configuration has a lower standard deviation than the rosette configuration.

For the high-energy samples, the average absorbed energy was 114.0 J ± 8.96 J for the proposed configuration, and 107.0 J ± 12.44 J for the rosette configuration. NISTIR 8145 (see Table 17 in [23]) states an average energy of 111.63 J ± 13.83 J.

### 5.2 Comparisons of Maximum Force Values

With regard to force values, the mean maximum force for the low-energy specimens was 34.5 kN ± 0.39 kN (where the uncertainty represents twice the standard deviation) for the proposed configuration, and 31.6 kN ± 0.83 kN for the rosette configuration. NISTIR 8145 (see Table 15 in [23]) states 31.9 kN ± 1.51 kN for the low-energy specimens, which is in better agreement with the rosette configuration.

The mean maximum force for the high-energy samples was 25.2 kN ± 0.41 kN for the high-energy configuration, and 26.4 kN ± 0.27 kN for the rosette configuration. NISTIR 8145 found that the average maximum force for the high-energy samples was 26.59 kN ± 2.79 kN. Again, the rosette configuration is in closer agreement to the values in NIST IR 8145 (see Table 18).

**Fig. 9:**
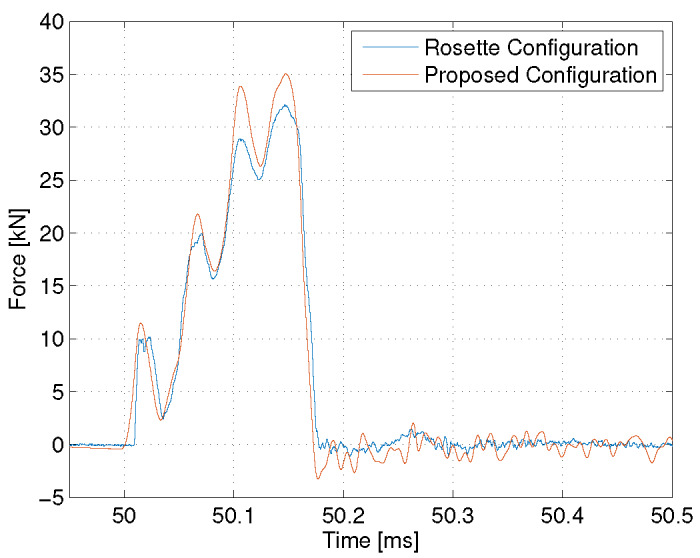
Force-time histories from the rosette configuration and proposed configuration strikers.

Typical time histories from both of the investigated strikers while breaking a low-energy sample (LL 137) are shown in Fig. 9. The amplitude of oscillation is larger for the proposed configuration and contributes to the measured larger peak force values. These oscillations may be mitigated in the proposed design by making the striker stiffer.

## 6 Summary

Design considerations for engineering improved instrumented strikers used for Charpy testing have been reported. Similar to other force transducers and certain other instrumented striker designs, the design presented here used a flexible area, referred to as the sensing element, which undergoes deformation and is instrumented to determine the force. With regard to mechanical geometry, design features have been given that prevent the sensing element from experiencing mechanical contact interference while undergoing deformation. Moreover, we have listed some design considerations to ensure that the strain field within the sensing element is free of stress concentrations. In considering the instrumentation and bridge circuit for the device, a configuration was given that yields a sensitivity that was experimentally shown to be mostly insensitive to impact location and distribution of forces. Although finite element results are not presented within this work, the proposed configuration also mitigates the response to certain vibrational modes (*e.g.*, modes that correspond to displacements in the x and z-axes) of the sensing element.

A prototype of the device was constructed and statically calibrated in a universal testing machine. The device demonstrated good repeatability, with most standard deviations lower than 1% for impact-location loading. The device was intentionally calibrated 2 mm and 3 mm away from the nominal impact location (a distance likely greater than what could occur in practice), and the deviations of the response at the force steps were less than 2% in most cases. Moreover, the device was calibrated by applying the force through three different test specimens of varying geometry and material. The device showed small sensitivity changes of less than 3% for these different specimens.

The device prototype was also used to break low- and high-energy samples. Good agreement was observed between the encoder energy and the instrumented striker energy. Although these design concepts have been implemented on a realized device, we do not claim that this device is optimal for instrumented testing. Currently, we are pursuing other ways to improve the instrument presented here. For instance, other strain gauge configurations may mitigate the response to off-axis forces and bending modes of the device even further. Alternatively, we are exploring customized strain gauges with widths greater than their length to attach to each side of the sensing element. Additionally, this paper has primarily covered static aspects of the device. We are also pursing designs that will reduce the differences between the static and dynamic sensitivity. This can be achieved by reducing the striker edge mass and moving the strain gauges closer to the front of the striker, where the impact occurs. Making the sensing element stiffer by increasing the width will also mitigate static and dynamic sensitivity differences. An anonymous reviewer pointed out that for testing and evaluation of future improvements to this device, one could monitor the response of each strain gauge using a quarter bridge circuit.

## 7 Appendix A: Dimensions of the Instrumented Striker

**Fig. 10:**
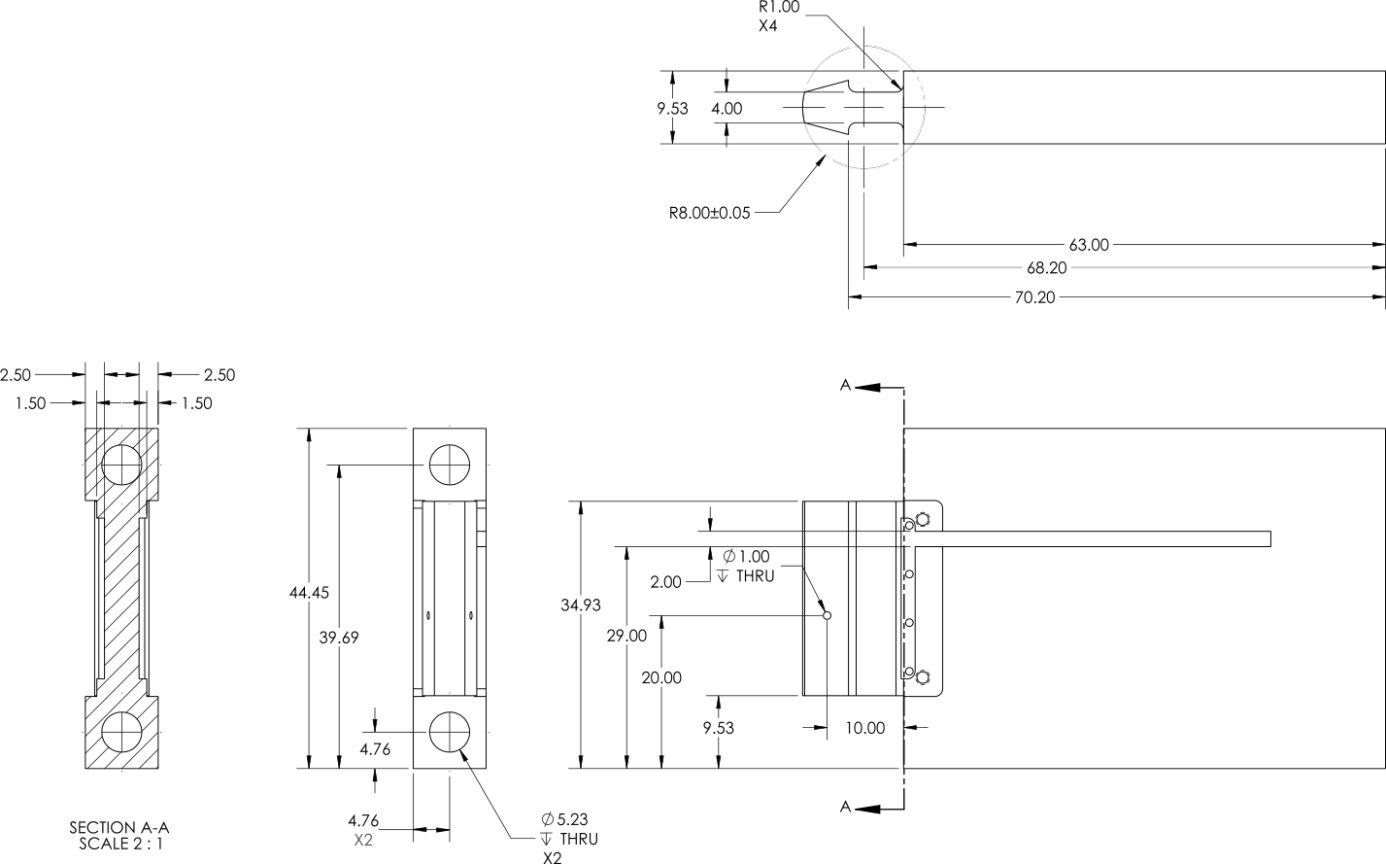
Dimensions (in units of mm) of the instrumented striker presented within this work.

**Fig. 11:**
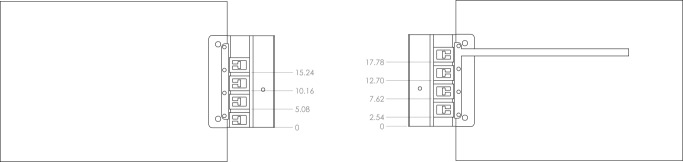
Placement of the Vishay SGK L1E strain gauges.
